# Organisational interventions in nursing care: A scoping review and descriptive system to support comparison

**DOI:** 10.1016/j.ijnsa.2026.100626

**Published:** 2026-07-10

**Authors:** Nienke Miedema, Martijn Felder, Dewi Stalpers, Catharina van Oostveen, Maureen Rutten-Van Mölken, Lucas Goossens, RN2Blend consortium

**Affiliations:** a Erasmus School of Health Policy & Management, Erasmus University Rotterdam, Rotterdam, the Netherlands; b Julius Centre for Health Sciences and Primary Care, Department of General Practice and Nursing Science, University Medical Centre Utrecht, Utrecht, the Netherlands

**Keywords:** Scoping review, Descriptive system, Nursing organisation, Causal inference, Interventions of (re)organising nursing care, Nursing research, Nursing workforce

## Abstract

**Background:**

The nursing profession across developed countries faces significant pressures, particularly due to nursing shortages, highlighting the need for effective organisational interventions of nursing care at hospital level, e.g. new nursing roles, experimenting with different types of nurses and differentiated practices of nursing, and the expansion of the nursing profession within the hospital. However, it is difficult to learn from those experiences, partly because of the absence of a standardised description of these interventions. We benefited from a window of opportunity that occurred after failed reorganisation attempts at national level in the Netherlands, leading to a wide range of local initiatives to experiment and organise nursing care differently.

**Objective:**

To develop a comprehensive descriptive system to systematically describe the diversity of interventions to reorganise nursing care in different hospital settings.

**Methods:**

A scoping review of Dutch grey literature on these initiatives was conducted to identify interventions to (re)organise nursing care in Dutch hospitals between 2015 and 2021. The results were analysed thematically and synthesised into a descriptive system, consisting of a checklist and a matrix, embedded in international literature to capture the diversity in the (re)organisation of hospital nursing care. We applied the descriptive system on two cases of interventions of (re)organising nursing care in Dutch hospitals.

**Results:**

Initially, 1102 records were identified, of which 27 were included in the review. Three main themes were determined: the organisation of different types of nurses, the organisation of different types of nursing work, and the organisation based on terms of employment. The three themes formed the basis for the descriptive system, each outlined with three dimensions describing each theme in more detail. Nine dimensions were identified within these three themes: (1) educational degree, competences, and subjective professional requirements, (2) patient care, indirect operational patient tasks, and quality and research, and (3) positions and embedding, quantity and ratios, and valuation. Additionally, the context was added to the descriptive system in order to take into account contextual factors not captured by the dimensions. All themes and dimensions proved valuable when applying the descriptive system to the two existing interventions.

**Conclusions:**

We propose a system of interventions of (re)organising nursing care to enable a systematic and comprehensive description of such interventions as the basis for evaluation.

**What is already known**

• Interventions to (re)organise nursing care are widely studied, but often lack consistent descriptions.
• Research on organisational interventions in nursing has been performed in many different contexts, on different scales, using diverse methodologies and terminology.
• It is difficult to establish how estimates of effectiveness can be transported across different settings.

Alt-text: Unlabelled box dummy alt text

**What this paper adds**

• This study proposes a descriptive system to harmonise detailed descriptions of intervention to (re)organise nursing care.
• The descriptive system defines three main themes: the organisation of different types of nurses; the organisation of different types of nursing work; and the organisation based on terms of employment.
• All themes consist of three dimensions each: (1) educational degree, competences, and subjective professional requirements; (2) patient care, indirect operational patient tasks, and quality and research; (3) and positions and embedding, quantity and ratios, and valuation.
• This descriptive system can support the evaluation of organisational interventions and their transferability to other settings and countries.

Alt-text: Unlabelled box dummy alt text

## 1 Introduction

Ageing of populations and substitution of secondary to primary care have led to increasingly complex, labour-intensive nursing care in hospitals. This complexity calls for a diverse and efficiently organised nursing workforce, the urgency of which became widely visible during the COVID-19 pandemic. Meanwhile, healthcare organisations are struggling with growing nursing shortages, resource constraints and high occupational turnover (Jacob et al., 2015; Saville et al., 2019). As a response to these developments, the organisation of the nursing profession is evolving in many ways: the range of nursing professionals is expanding, new roles are determined, experiments with different types of nurses and differentiated nursing practices are introduced, and boundaries within the current workforce shift or fade (Butler et al., 2019; Dubois and Singh, 2009; Griffiths et al., 2020; Saville et al., 2019). To help researchers and healthcare organisations learn from each other, these organisational practices must be evaluated in a generalisable or transferable manner.

Many studies have been conducted to assess a broad variety of organisational changes and interventions to (re)organise care within the nursing profession. Several studies concentrated on new nursing roles and expanding the nursing profession by substituting physicians with nurse specialists (Lovink et al., 2017a, 2017b; Silies et al., 2025; Tsiachristas et al., 2015). Other studies assessed the association between different nurse staffing levels and nursing care-related outcomes (Aiken et al., 2018; Blume et al., 2021; Dietermann et al., 2021; Milstein and Schreyoegg, 2020; Morioka et al., 2026; Nwanosike et al., 2026). A third field of research focussed on different combinations of skills and nursing practices within the profession and their effects on various outcome measures such as mortality, quality of care and turnover (Aiken et al., 2017; Brady et al., 2025; Staggs and Dunton, 2012; Twigg et al., 2012; Yang et al., 2012).

Although these papers made important contributions to identify potential effects of organisational interventions, it has also been observed that these interventions may have been operationalised in different ways, performed in many different contexts and implemented on different scales (Butler et al., 2019; Cunningham et al., 2019; Dubois and Singh, 2009; Griffiths et al., 2020; Saville et al., 2019; Simon et al., 2014; Spilsbury and Meyer, 2001). For instance, Cunningham et al. (2019) noted how the concept of ‘skill mix’ is used in many different ways in literature, and developed a conceptual model to explain how it can have one or multiple interdependent dimensions (Cunningham et al., 2019, p. 261).

These issues make it difficult to see to what extent research findings are meaningful in specific contexts. The problem can be clarified further using two distinct concepts from causal inference theory: effect modification and consistency. *Consistency* refers to the completeness of the description of an exposure (Hernán, 2016; Rehkopf et al., 2016). It can be defined informally as: ‘Have I defined the exposure to include all causally relevant features?’ (Cole and Frangakis, 2009). More formally, causal effects are not well defined if the exposure can be applied in more than one way and different versions of the exposure lead to different outcomes (Hernán, 2016). Applied to a study on the effect of increased nurse staffing levels, for instance, the consistency condition could require that researchers not only report the number of nurses but also define the tasks, skills, and responsibilities of the current and additional staff. *Effect modification* recognises that ‘the’ causal effect does not exist (Hernán and Robins, 2020). The effect of an exposure (in this case an intervention to (re)organising nursing care) is modified by the context in which it is applied, e.g., the characteristics of the population under study, the starting position, hospital-characteristics and other aspects like care processes, management support, or the professional conduct framework of an organisation or country.

The combination of the two concepts highlights that it is crucial to describe both the intervention and the context in a sufficiently detailed manner when the effects of an organisational intervention are evaluated. However, without a standard for these detailed descriptions, it is difficult to see whether all relevant aspects are considered. The objective of this study was therefore to develop a comprehensive system to describe the diversity of interventions to (re)organise hospital nursing care. As an illustration of its usefulness, we applied it to two cases of interventions to reorganise nursing care.

## 2 Methods

The descriptive system was developed and applied in three phases. First, we performed a scoping review of the documentation of innovations in the Dutch hospital nursing landscape. Secondly, the results of this scoping review formed the foundation of the descriptive system. To ensure its relevance across different counties, we embedded it in international literature. In the final step, we used two real life cases of nursing interventions in Dutch hospitals to test and apply the usability of the descriptive system.

### 2.1 Scoping review

We conducted a scoping review based on the framework described by Arksey and O’Malley (2005). The protocol contains the following five stages: (1) identifying the research question; (2) identifying relevant studies; (3) study selection; (4) charting the data; and (5) collating, summarising, and reporting the results. Below, we further describe steps taken during these stages.

#### 2.1.1 Identifying the research question

Between 2016 and 2019 a national legislative initiative was in development to (re)organise skill mix and differentiated practices of nursing. This initiative ultimately failed to result in the planned legislative changes in 2019. However, during and after this period, hospitals in the Netherlands were incentivised to reorganise their nursing workforce (Schalkwijk et al., 2024) and started to experiment with a new mix of nursing skills and/or differentiated practices of nursing (Van Kraaij et al., 2022). Because the Dutch healthcare system is highly decentralised, this led to a wide variety of reorganisations. Hospitals were keen to learn from one another and produced an extensive amount of ‘grey literature’ with descriptions of past, current, and projected nursing practices and their reorganisation. Therefore, a unique opportunity arose to perform a scoping review of this grey literature to inform the descriptive system of interventions to (re)organise nursing care. We benefited from this window of opportunity in the Dutch hospital landscape in this turbulent period of increased experimenting and reorganising the nursing workforce. The scoping review was based on Dutch grey literature produced by actors involved in the (re)organisation of nursing care within Dutch hospitals. These actors included health care organisations such as hospitals, nurses’ unions, healthcare research institutes, educational institutes, Dutch professional nursing journals, the Ministry of Health, government agencies as well as consultancies.

#### 2.1.2 Identifying relevant studies

Systematic searches were conducted from 2015 up to December 2021 using various electronic databases including those managed by Dutch nursing organisations, Dutch nursing journals and Dutch hospital organisations. This period was chosen in order to include all literature produced around the failed legislative initiative between 2016 and 2019. An initial limited search strategy was conducted using Dutch terminology to gain familiarity with reports and papers. All documents were analysed by NM for keywords and terms that should be added to those used during the initial search strategy. Search results were calibrated by discussing them with two other researchers (LG and MF). The final search strategy can be found in appendix A. Two additional steps were taken to increase document saturation. All identified reports and articles were scanned for additional sources that could be relevant. Additionally, digital Dutch conferences were identified and followed for subsidiary identification of relevant projects. Furthermore, the research team contacted existing networks and relevant organisations. The Dutch Association of Hospitals (NVZ) and the Dutch Federation of Academic Hospitals (NFU) provided relevant developments, projects, experiments, and implementations of differentiated nursing practices in the Netherlands between 2015 and 2021.

#### 2.1.3 Study selection

**Fig. 1:**
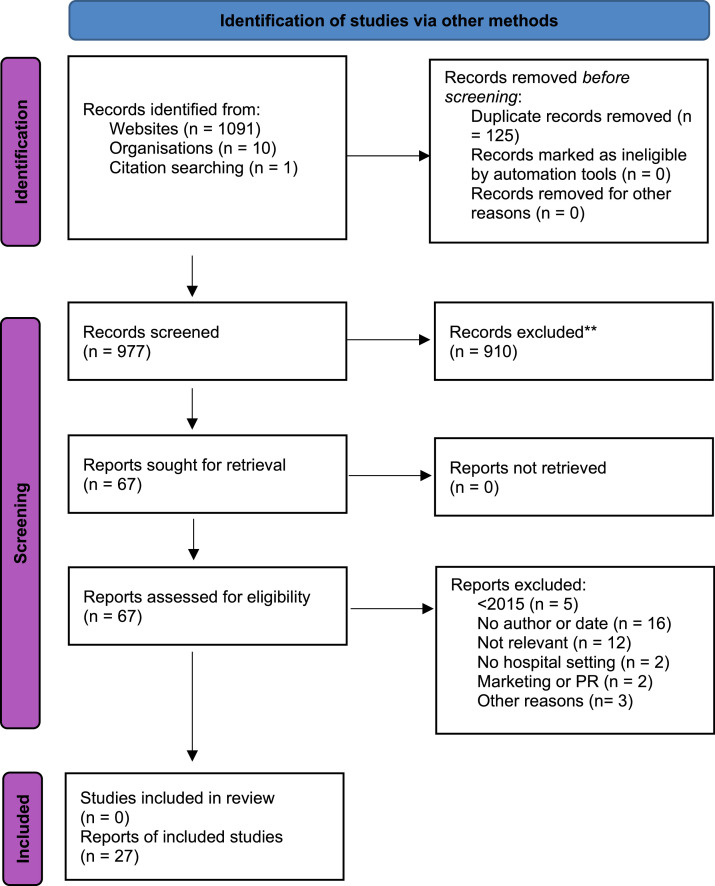
PRISMA flow diagram.

We identified 1102 potentially relevant data sources. After removing duplicates, 977 data sources remained. The first author screened the title and abstract of these sources, using one specific inclusion criterion; (1) sources needed to describe the (re)organisation of nursing care within Dutch hospitals. After this initial screening, 67 data sources were retrieved. The eligibility of these sources was discussed amongst the remaining authors. The final inclusion criteria included: (2) project reports describing experiments, and/or implementation of organisational nursing interventions; (3) pertaining to hospital nursing workforce; and (4) national documents describing overarching and more theoretical implementation of interventions to (re)organise nursing care. After this, reports (1) in other settings than hospitals; (2) before 2015 or after 2020 (start of COVID); (3) containing marketing or PR sources; and (4) without an author or date were excluded from further analysis. In the end, 27 sources were identified and included in the analysis (see also the PRISMA flow diagram in Fig. 1).

#### 2.1.4 Charting the data

Most sources identified were institute reports (n=10) and professional literature (n=7). In addition, several research reports were included (n=5). Lastly, working group reports (n=2), a programme report (n=1), a policy report (n=1) and a legislative proposal (n=1) were included in the scoping review. Appendix E displays an overview of the 27 data sources used in the scoping review.

The data-extraction chart consisted of the following information: citation/type of source, year of publication, type of document, aim/objectives, focus of report, and key findings regarding the interventions of (re)organising nursing care.

#### 2.1.5 Collating, summarising, and reporting the results

The literature was analysed thematically through a four-step process (cf. Arksey and O’Malley, 2005). In a first step, open coding was applied to all data sources. We identified all text fragments that referred to the organisation of nursing care to ensure that the diversity of organisational interventions could be adequately captured. As a second step, these individual text fragments were reviewed, compared and arranged into thirty distinct elements that mattered for understanding the nature of (re)organising nursing care. In a third step, these elements were grouped together into nine distinct dimensions of (re)organising nursing care. In the last step, we grouped these nine dimensions into three overarching themes. The initial coding and identification of text fragments was initiated by the first author, after which the grouping of these text fragments into elements, dimensions and themes was discussed amongst all authors. Appendix B gives an overview of the analytical steps taken to work from text fragments to overarching themes.

### 2.2 Descriptive system

Based on the nine dimensions and three overarching themes from our scoping review of the Dutch grey literature we developed a descriptive system that can support the evaluation of organisational interventions and their transferability across settings and countries. To that end we translated the underlying elements of the dimensions into internationally relevant terminology by connecting them to the descriptions and wording used in six relevant international studies regarding organisational changes and developments within the nursing profession (Butler et al., 2019; Dubois & Singh, 2009; Griffiths et al., 2020; Saville et al., 2019; Spilsbury & Meyer, 2001; Cunningham et al., 2019). These studies were identified in the first stage of the scoping review (identifying the research question) and were selected based on the following criteria: (1) reviews; (2) hospital nursing care; and (3) discussing models or interventions of (re)organising nursing care.

The initial descriptive system was validated in discussions with experts in the Dutch nursing practice and its usefulness was tested in three focus groups with nurses, nurse-managers, general hospital managers, and policy officers.

This process resulted in the final descriptive system containing three overarching themes with three underlying dimensions each. We tested the descriptive system on two specific cases representing two different organisational interventions. One case was about the introduction of nursing and the other was a pilot experimenting with care teams. The cases differed from one another to allow for a testing of the descriptive system and its various components. We selected these cases purposively based on the following criteria: (1) they involved the reorganisation of nursing; (2) they differed from one another to allow for a testing of the descriptive system and its various components; and (3) we had access to enough information about these cases to fill out the descriptive system.

## 3 Results

### 3.1 Themes and dimensions

#### 3.1.1 Organising different types of nurses

The first theme that emerged centres around differentiating between different types of nurses. It is important to understand that in several countries, including the Netherlands, there are different educational pathways to become a registered nurse, both at the vocational level and at the baccalaureate level. Even though Dutch registered nurses can have very different training backgrounds, they tend to do similar work as soon as they start practicing as a hospital nurse. Nurse managers hypothesised that this demotivates the bachelor educated nurses. Many Dutch hospitals therefore sought to distinguish practices more clearly between vocational and bachelor-trained nurses (Van Kraaij et al., 2022). Our scoping review shows they did so along three distinct ways: educational qualifications, competences, and professional growth and disposition.

##### 3.1.1.1 Educational qualifications

Sixteen sources described educational qualifications as a way to organise nursing personnel (Berkhout, 2019; Commissie Meurs, 2019; de Vos et al., 2017; de Vos and van Opstal, 2015; Deggens et al., 2018; Geerts et al., 2015; Hamel, 2019a; Lambregts et al., 2015; Martini et al., 2021; Peters, 2020a; Peters and Alem, 2017; Pool et al., 2020; Secchi, 2017; Stalpers, 2017; van Lieshout et al., 2018; Van Schothorst-van Roekel et al., 2019). Nursing educational degrees are based on specific educational profiles displaying knowledge, skills and attitudes (Lambregts et al., 2015; Stalpers, 2017). These educational profiles show a certain hierarchy embedded in the responsibilities and competences that are associated with the level of education.

In addition to the different levels of education, several sources described post-degree specialisation as an important element to further distinguish different nursing positions within an organisation (de Vos and van Opstal, 2015; Peters, 2020a). Post-degree specialisation includes a master’s degree, such as an Advanced Practice Nurse or a Physician Assistant. These educational profiles are clearly defined and embedded in national or even international legislation and accreditation. It also included other post-degree education, which is often less clearly defined (Pool et al., 2020). Post-degree educational requirements can be based on care specifications for a specific patient group, such as diabetes care, or based on the location where the care is performed, such as working on specialised hospital wards in an Intensive Care Unit (ICU) or an Emergency Room (ER). The educational courseload can differ greatly in post-degree education.

Hospital nursing teams can be organised based on these various levels of education, assigning specific responsibilities and competences to specific nursing groups on the work floor. Educational qualifications can provide the backbone of specific skill- or staff-mix structures by differentiating nursing practices.

##### 3.1.1.2 Competences

Eighteen sources described nursing competences as another approach to organise different nursing positions within the workforce (Berkhout, 2019; de Vos et al., 2017; de Vos and van Opstal, 2015; Deggens et al., 2018; Gakes et al., 2018; Galama et al., 2019; Geerts et al., 2015; Hamel, 2019b; Lambregts et al., 2015; Martini et al., 2021; Meijerink, 2017; Paardekooper, 2020; Peters, 2020b; Peters and Alem, 2017; Pool et al., 2020; Schroder, 2018; Stalpers, 2017; van Lieshout et al., 2018). Competences can for instance be defined as knowledge, insight, attitude, capabilities, and skills needed to respond accordingly to specific professional circumstances (Galama et al., 2019; Lambregts et al., 2015). Several sources referred to the Canadian Medical Education Directions for Specialists (CanMEDS) as a specific competency-based organisational method (de Vos et al., 2017; de Vos and van Opstal, 2015; Geerts et al., 2015; Lambregts et al., 2015; Peters and Alem, 2017; van Lieshout et al., 2018; Van Schothorst-van Roekel et al., 2019). The CanMEDS model describes nursing competences in seven distinct roles: the nurse as health care provider, communicator, collaborator, reflective Evidence Based Practice (EBP) professional, health promotor, organiser, and promotor of professionalism and quality (Lambregts et al., 2015). Each role requires nurses to display specific levels of knowledge, skills, and attitudes to show they are fulfilling the competence. Another competency-based method referred to was the Entrustable Professional Activities (EPA) (Pool et al., 2020). Both methods can be used to enforce specific levels of knowledge, skills, and attitudes to demonstrate a nurse is competent and qualified on a specific level. These levels are often embedded in professional profiles, educational profiles, or specific job profiles, which are used to define and organise different groups of nurses.

#### 3.1.2 Professional growth and disposition

Several sources discussed additional criteria beyond educational qualifications and competences to organise different groups of nurses. These additional criteria can be defined as aspects of professional growth and disposition, such as personal portfolios, autonomy, career ambitions, personal experiences and intrinsic motivation (de Vos and van Opstal, 2015; Deggens et al., 2018; Galama et al., 2019; Geerts et al., 2015; Hamel, 2019b; Martini et al., 2021; Peters, 2020a; van der Velde et al., 2019). Hamel (2019b) described an example of these more soft demands in processes of developing a new nursing role within hospitals. The assessment of functioning in this new role should not just be based on objective preconditions, such as educational-based requirements, but also on the personal experience and intrinsic motivation of an individual (Hamel, 2019b). Another source described several aspects of professional growth and disposition within their job profiles, such as knowledge, social skills, influence, independence, responsibility, communication and vigilance (Deggens et al., 2018).

#### 3.1.3 Organising different types of nursing work

The second theme that was prominently featured in the grey literature revolved around the different types of nursing work that different types of nurses (theme 1) are supposed to do. Differentiations were made in terms of patient care, indirect operational patient tasks, and quality and research.

##### 3.1.3.1 Patient care

Nine sources described different patient-care-related tasks as a means to organise nursing care (de Vos and van Opstal, 2015; Galama et al., 2019; Geerts et al., 2015; Hamel, 2019b; Hillebrand et al., 2019; Martini et al., 2021; Peters, 2020a; Peters and Alem, 2017; van Lieshout et al., 2018). Martini et al. (2021) described how the organisation of nursing activities evolved from task-based patient care to team-based patient care. In the ’80s, nurses were more restricted in their assigned duties. For example, a nurse assigned to medicine duties was not allowed to comfort a crying patient. Today, many hospitals organise their nursing workforce in teams that share responsibilities and tasks according to preferences, daily demands, and individual abilities (Martini et al., 2021).

Several sources also discussed complexity of care as the basis of organising patient care (Galama et al., 2019; Geerts et al., 2015; Hamel, 2019b; Hillebrand et al., 2019; Peters, 2020a; Peters and Alem, 2017; Schroder, 2018; van Lieshout et al., 2018). Two sources further defined complexity of care using two criteria: case complexity and patient complexity (Geerts et al., 2015; van Lieshout et al., 2018). Additionally, an institute report describing new nursing positions within Peters and Alem (2017) suggested a different term: predictability of care. Both sources - using complexity of care - distinguish five items of case complexity and five items of patient complexity which both determine a “complexity system” (Geerts et al., 2015; van Lieshout et al., 2018). Case complexity is described as aspects on a more systematic level, e.g. the complexity of disease or the complexity of the care delivery system in terms of for example high intensity multidisciplinary care teams. Patient complexity is described as aspects on a more personal level, e.g. vulnerability of the individual and complex care needs that span multiple care domains (van Lieshout et al., 2018, p. 13).

##### 3.1.3.2 Indirect operational patient tasks

Multiple sources described indirect operational patient tasks in addition to patient care to organise nursing care (Berkhout, 2019; de Vos et al., 2017; de Vos and van Opstal, 2015; Deggens et al., 2018; Martini et al., 2021; Paardekooper, 2020; Peters, 2020a; Peters and Alem, 2017; Van Schothorst-van Roekel et al., 2019). Several definitions can be found to describe indirect operational patient tasks, such as invisible nursing work (e.g. coordination of care, coaching of team members), care transcending tasks (e.g. professionalisation of the work environment) or management activities (Berkhout, 2019; Martini et al., 2021; Van Schothorst-van Roekel et al., 2019). All tasks and activities were described as care tasks indirectly related to the care of patients in the nursing unit (Berkhout, 2019; de Vos et al., 2017; de Vos and van Opstal, 2015; Deggens et al., 2018; Paardekooper, 2020; Peters, 2020a; Peters and Alem, 2017; Van Schothorst-van Roekel et al., 2019). The nature of these care activities is vital for nursing work when caring for patients, and perhaps even more so in the organisation of a nursing team. These additional tasks ensure the functionality of the nursing team and a steady operation of the workplace. The formal or informal distribution of these tasks can lay the foundations for the team structure.

##### 3.1.3.3 Quality of care and research

Several sources described quality of care and scientific research as an additional aspect to organise different nursing positions (Hamel, 2019b; Van Schothorst-van Roekel et al., 2019). Quality of care can be described as “initiating and monitoring quality of nursing care, and the ability to take action if needed” (Peters and Alem, 2017, p. 28). Examples of tasks that are included in quality of care are guarding safety and regulation, reflecting and feedback, participating in committees, updating protocols, implementing new guidelines (de Vos and van Opstal, 2015; Lambregts et al., 2015; Peters and Alem, 2017). Additionally, research tasks were identified in several sources (Commissie Meurs, 2019; Lambregts et al., 2015; van der Velde et al., 2019). Research tasks can be described as “initiating and collaborating in practical and scientific research” (Peters and Alem, 2017, p. 29). Both tasks can be used to further organise and differentiate the nursing profession.

#### 3.1.4 Terms of employment

The third theme - terms of employment - provided the organisational and institutional conditions that enable and stabilise the differentiations in nursing roles and work organisation. This theme captured how hospitals formalised and embedded these structures through staffing policies, employment arrangements, and recognition systems. As such, it brings together the previous themes by highlighting the organisational levers used to operationalise and sustain differentiated nursing practice. Within this theme, we distinguish between three dimensions: positions and embedding, quantity and ratio, and recognition and reward.

##### 3.1.4.1 Positions and embedding

Two types of nursing positions are identified in the grey literature: nursing profiles and nursing roles. The term profile typically refers to the formal embedding of nursing positions within organisational and regulatory frameworks, whereas role points to more informal, process-oriented forms of differentiation. Seventeen sources used the concept of profile to describe how nursing positions are structured and embedded (Commissie Meurs, 2019; de Vos et al., 2017; de Vos and van Opstal, 2015; Deggens et al., 2018; Gakes et al., 2018; Galama et al., 2019; Geerts et al., 2015; Hamel, 2019a; Jackson, 2015; Lambregts et al., 2015; Meijerink, 2017; Paardekooper, 2020; Peters and Alem, 2017; Schroder, 2018; Stalpers, 2017; van der Velde et al., 2019; van Lieshout et al., 2018). Three types of profiles can be distinguished: the professional profile, which outlines the nursing workforce at a national level; the educational profile, which defines training requirements in line with national and/or European directives; and the job profile, which specifies position-specific responsibilities at the level of individual organisations (Lambregts et al., 2015).

In contrast, the concept of nursing role is used in several sources to describe how nurses and their work are differentiated in practice, often without formal policy (de Vos et al., 2017; de Vos and van Opstal, 2015; Deggens et al., 2018; Lambregts et al., 2015; Martini et al., 2021; Paardekooper, 2020; Peters, 2020a; Peters and Alem, 2017; Scheer and Westerbeek, 2021; van Lieshout et al., 2018; Van Schothorst-van Roekel et al., 2019). While the term lacks a shared definition, it is used to express distinctions based on combinations of responsibilities, tasks, behaviours, and requirements (de Vos and van Opstal, 2015; Deggens et al., 2018). Paardekooper (2020), for example, employed the term to distinguish between two nursing functions that were not yet formalised within organisational decision-making processes.

##### 3.1.4.2 Quantity and ratios

Multiple sources described the arrangement of the workforce as a specific mix of nursing quantities or ratios combining a specific number of patients with nurses (Commissie Meurs, 2019; Deggens et al., 2018; Gakes et al., 2018; Galama et al., 2019; Hillebrand et al., 2019; Paardekooper, 2020; Secchi, 2017; Stalpers, 2017; van der Velde et al., 2019; Van Schothorst-van Roekel et al., 2019). A mix of nursing positions is defined as “the numerical ratio between different types of nursing positions” (Galama et al., 2019; Hillebrand et al., 2019). Several sources expressed a desire to find the optimal mix of nursing positions (Deggens et al., 2018; Gakes et al., 2018; Paardekooper, 2020; Secchi, 2017). Another example, a labour market research report, assessed multiple perspectives of introducing new professional profiles. Each perspective includes a scenario describing a future mix of nursing positions based on new professional profiles (van der Velde et al., 2019).

##### 3.1.4.3 Recognition and rewards

Several sources indicated methods of recognition and rewards as a component when organising nursing positions (Commissie Meurs, 2019; Galama et al., 2019; Hamel, 2019b; Hillebrand et al., 2019; Paardekooper, 2020; Peters, 2020a; Pool et al., 2020; Scheer and Westerbeek, 2021; van der Velde et al., 2019; van Lieshout et al., 2018; Van Schothorst-van Roekel et al., 2019). Sources characterised rewards from two different perspectives. The first perspective is purely economical, the monetary rewards of different nursing positions, which can be defined as: “the valuation of classifying positions in a ranking order, based on the relative level of job complexity” (Galama et al., 2019, p. 87). For example, additional tasks and responsibilities within a nursing position valuad with an increase in salary (Peters, 2020a). Van Lieshout et al. (2018) described valuation as an important and influential aspect for the acceptance and implementation of new ways of organising nursing care.

The second perspective described rewards - or recognition - as additional methods to appraise and stimulate nurses within the organisation (Commissie Meurs, 2019; Galama et al., 2019; Martini et al., 2021; Peters, 2020a). Examples of these methods are investing in educational opportunities and creating time for research opportunities (Commissie Meurs, 2019; Martini et al., 2021). Other sources discussed the term “career opportunities” without specifying a clear definition.

##### 3.1.4.4 Context

While the three themes and their underlying dimensions offer a structured framework for understanding the organisation of nursing types, work, and employment conditions, several sources stress that organisational interventions of nursing care cannot be fully interpreted without considering prerequisite contextual factors. Four reports (Deggens et al., 2018; Galama et al., 2019; Hillebrand et al., 2019; Paardekooper, 2020) highlight how nursing organisation is shaped by dynamic, context-specific conditions that fall outside the identified themes while still impacting nursing implementation and possible outcomes.

These contextual conditions can be grouped into three levels: patient-level, organisational-level, and country-level context. Importantly, these are not analytical categories of nursing work itself, but external conditions that shape how the organisation of nursing types and tasks can or cannot take place in practice.

At the patient level, context refers to specific patient traits that could influence the organisation and implementation of nursing care. For example, most hospitals cluster care delivery based on patient groups with similar diagnoses or treatment pathways. These clusters then influence the deployment of nursing teams and the delineation of roles within them (Paardekooper, 2020). Thus, nursing organisation varies depending on the type of patient population served, regardless of the formal organisation model applied.

At the organisational level, factors such as existing team composition, intergenerational or cultural dynamics, workplace architecture, and specific workstyles may facilitate or constrain the adoption of particular nursing practices (Deggens et al., 2018; Hillebrand et al., 2019). These are not choices within the design of nursing work, but rather the contextual conditions that pre-structure what is possible or likely.

Hillebrand et al. (2019) discussed the nursing labour market, including the inflow and outflow of professionals. Other important contextual factors — such as the type of health and social care system, reimbursement models, provider payment mechanisms, access to care, nurse-to-population ratios, healthcare expenditures, and cultural influences — were briefly mentioned but largely unexplored.

### 3.2 The descriptive system

The descriptive system consists of a checklist based upon the themes and dimensions synthesised from the scoping review, providing a systematic walkthrough of all relevant elements of (re)organisational interventions of nursing care. The checklist provides four questions for each dimension to identify and outline the important aspects of the current organisation of nursing care (question 1 and question 2, describing the baseline), and important aspects of the intervention to change the future organisation of nursing care (question 3 and question 4, describing the organisational aspects that were newly introduced or changed). If applicable, they should be defined or described in sufficient detail for the context of a particular study. An overview and additional details are provided in a written, coherent summary of 300-600 words at the end of the checklist. The checklist can be found in appendix C.

The filled-out checklist can be summarised in a matrix overview that provides a schematic summary of two states of nursing organisation: the current organisation of nursing care and the interventional change or addition to the current nursing organisation (see appendix D).

We illustrate the application of the descriptive system with two cases of changes in the organisation of nursing care. For each case, the checklist was completed and summarised into the matrix, facilitating a standardised comparison between the two organisational interventions. Based on these matrices, we were able to write the following coherent summaries.

### 3.3 Case 1. Introducing a new nursing role, the nurse coordinator

This case concerns the introduction of a new nursing profile, the nurse coordinator, in a large regional teaching hospital’s gastrointestinal surgical ward. The nursing staff in this ward consists of three groups based on educational background: vocational, bachelor’s, and master’s educated nurses. Despite these differences, all nurses shared the same nursing profile, salary scale, and employment conditions prior to the intervention. Nursing positions were embedded within the CanMEDS framework, which guided role expectations and competencies. The ward operated a team-based nursing organisation with nurse-to-patient ratios ranging from 1:4 to 1:6. Indirect operational patient tasks were formally embedded within nursing roles, but leadership and coordination responsibilities were informally assigned or only visible in management functions. Career progression opportunities and formal leadership roles within the nursing team were limited.

The intervention consisted of introducing the nurse coordinator position, accompanied by a newly developed nursing profile and a distinct, higher salary scale. This role was designed to enhance career pathways and foster professional development. Recruitment focused on internal candidates’ competencies, intrinsic motivation, ambition, and practical experience rather than educational level. Five nurse coordinators were appointed.

The nurse coordinators focused on coordinating nursing care, coaching and supporting the team, professionalising the workplace, and managing care delivery. Over the course of one year, this role facilitated the implementation of a newly introduced classification system for patient complexity, a new nursing work structure, and redesigned physicians’ ward rounds. By formalising leadership roles that were previously done informally, the nurse coordinator strengthened team functioning and clarified role boundaries.

Contextual factors influenced the intervention’s development and implementation. The ward served adult patients that underwent gastrointestinal surgery, with a bed capacity that fluctuated between 13 and 19 depending on staffing levels and workload. Patients varied widely in age, complexity of care and surgical interventions. Patient admissions were planned according to surgical schedules but were frequently disrupted due to staff shortages, leading to bed closures. These organisational challenges reflect broader national trends of nursing shortages and high turnover. Additionally, the implementation of the nurse coordinator was politically sensitive, as differentiation based on educational background or other criteria remains a contentious issue within Dutch nursing practice.

**Fig. 2:**
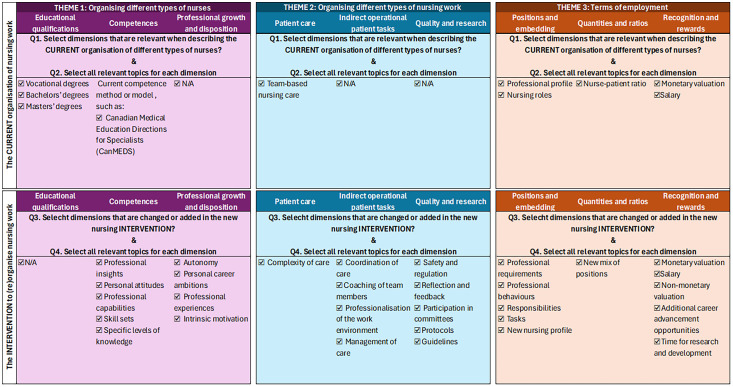
The filled-out matrix of the descriptive system for case 1 (Introducing a new nursing role, the nurse coordinator).

The checklist for case 1 can be found in appendix F and the matrix is shown in Fig. 2.

### 3.4 Case 2. Working with more students: introducing nursing care teams

This case describes a pilot study, introducing a care team in a trauma surgical ward of a regional teaching hospital. The ward provides care for adult patients with varying levels of complexity, admitted for surgical interventions following traumatic injuries. The nursing team is composed of vocational, bachelor’s, and master’s educated nurses. Two nursing profiles exist, i.e. staff nurse and nurse coordinator, each linked to educational qualifications and corresponding individual salary scales. Nursing organisation was primarily individual team-based, with nurses caring for their own patients and supporting colleagues as needed. The nurse-to-patient ratio was approximately 1:4, with surplus nursing students present. These surplus students were considered to be in training and were not assigned patient-responsibility or designated tasks.

Nationally, the Dutch nursing workforce faces shortages and high turnover, driving this hospital initiative to explore sustainable staffing and educational approaches. The pilot introduced a new organisational model where one graduated nurse works together with two to three nursing students in a care team responsible for six patients during the day shift, increasing the nurse-to-patient ratio to 1:6. After an onboarding period, students actively participated in hands-on patient care according to their academic level and year. The graduated nurse coordinated patient care, delegated tasks, and supports the students’ learning. A list of non-core nursing tasks was developed and delegated to nursing students and nursing aides, creating a task-based nursing organisation model.

This model aimed to sustain care delivery with fewer qualified nurses while enhancing educational opportunities for students, who benefited from a more structured learning environment compared to surplus student roles. The care team model also clarified delegation of tasks and reinforced the nurse coordinator’s role as educator and care coordinator.

Contextually, the pilot was conducted on a 12-bed ward within a hospital undergoing frequent relocations due to shortages of nurses and an approaching move to a new hospital building. The nursing team was highly motivated and actively involved in the pilot’s organisation despite the challenges of frequent moves.

**Fig. 3:**
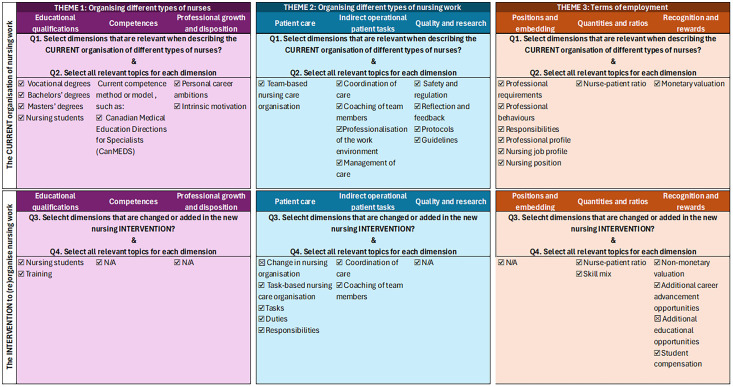
The filled-out matrix of the descriptive system for case 2 (Working with more students: Introducing nursing care teams).

The checklist for case 2 can be found in appendix G and the matrix is shown in Fig. 3.

## 4 Discussion

We conducted a scoping review of grey literature with the aim of developing a descriptive system that allows for better comparison of interventions of (re)organising nursing care across hospitals and countries. The large variation when describing interventions of (re)organising nursing care in the literature can create problems when assessing the effectiveness of different organisational interventions and transferring them to other contexts. To our knowledge, this is the first attempt to create a standardised description of organisational interventions in nursing in such a detailed manner. The use of our descriptive system, which includes both the checklist and the matrix that summarises the information from the checklist, can support the comparison of different interventions in evaluation studies. We have identified three distinct themes that were driving the nature of the organisational interventions (1) organising different types of nurses; (2) organising different types of nursing work; and (3) terms of employment. Within these three themes, we identified various dimensions such as educational degrees, competences, positions and ratios.

The descriptive system that was based on these themes and dimensions showed its usefulness in two cases, improving consistency and taking into account possible effect modification. The first case described the introduction of a new role, which was labelled ‘nurse coordinator’. Although this label may seem informative, it does not consistently describe what the role entails, nor how it affects the terms of employment already in place in the hospital. Specific details are needed to be able to understand what this intervention and its observed effects can teach us about the organisation of nursing workforces across hospitals and countries. After all, the requirements and responsibilities of nurse coordinators in relation to other healthcare workers might greatly differ. For example, the new position could potentially be filled by both a bachelor or a vocational nurse. The position, in this case, was filled by existing team members, which is also relevant. Taking into account these elements improves consistency.

The second case described a pilot experimenting with care teams. Similar to the first case, this label seems to inform readers only to some extent. In this case, the effect of introducing such a care team - consisting of students or nursing aides - will differ greatly from, for example, a team of more nurses or other certified health care professionals. It also depends on the way the teams works. Although the introduction of these teams may seem to imply the introduction of team-based working, this was not the case, as team-based working was already practiced in this ward. In fact, the intervention introduced some aspects of task-based nursing. The effect of introducing a nurse coordinator or a care team is modified by contextual factors, such as the patient group or the size of the ward. The organisation of a surgical ward and its patients may be more structured and protocol-based than a non-surgical ward.

All dimensions proved valuable in describing the two cases, although not all options within dimensions were applicable. This does not imply that these options are not valid, just that they were not affected by the change that was introduced or not described. An unobserved dimension may be just as informative as an observed dimension for a researcher defining the organisational nursing intervention.

The principle that context matters has often been recognised, for instance by Buchan et al. (2015) and Cunningham et al. (2019). In addition, the RN4CAST consortium showed that in-hospital mortality in specific patient groups can vary even more within countries than between countries (Aiken et al., 2018). This underlines that ‘context’ refers to much more than just country. Furthermore, even seemingly precise descriptions of organisational interventions can sometimes be improved. For example, when nurse ratios are calculated and organisational interventions are defined as adding an additional registered nurse (Aiken et al., 2017, 2014; Ball et al., 2018): who is this additional nurse and what do they do? Nurses can vary in educational levels (theme 1, dimension 1) based on country specific differences (van Kraaij et al., 2023). Additionally, the work carried out (theme 2, dimension 4 and 5) and competences held (theme 1, dimension 2) by this nurse can also vary (Stalpers et al., 2022).

### 4.1 Limitations

The focus on Dutch grey literature could limit the exhaustiveness of our descriptive system. We attempted to mitigate this limitation by relating and connecting our findings to six international studies on organisational interventions of nursing care. No dimensions or themes identified in the grey literature were directly in conflict with the international studies. However, we did add some topics to the descriptive system that were only observed in the international literature to further illustrate the scope of the dimensions. For example, shift patterns are not common in the Dutch health care system but are often included in health care systems in other countries (Butler et al., 2019). It is therefore possible that there are unobserved organisational factors that could potentially impact the robustness of our findings.

These limitations were considered when constructing the descriptive system. Each dimension provides a broad interpretation of the concept, which makes it possible to include possible unobserved organisational factors. The downside of this might be that it leads to less consistent descriptions which reduce the possibility to make causal claims. This last limitation highlights the need for more studies and analysis to strengthen the understanding and reporting of organisational interventions of nursing care in future research.

### 4.2 Conclusion

The descriptive system developed in this paper, consisting of a checklist that is synthesised into a matrix of three themes and nine dimensions, makes it possible to describe interventions to organise nursing care in detail, as was illustrated by the cases introducing a nurse coordinator and nursing teams including student-nurses. This is important for two reasons: firstly, to better understand the differences between organisational interventions that seem similar at first glance, and secondly, for the evaluation of organisational interventions in nursing care provided in hospital settings.

## Funding sources

This work was supported by Dutch Ministry of Health, Welfare and Sports.

## CRediT authorship contribution statement

**Nienke Miedema:** Writing – review & editing, Writing – original draft, Visualization, Validation, Software, Resources, Project administration, Methodology, Investigation, Formal analysis, Data curation, Conceptualization. **Martijn Felder:** Writing – review & editing, Writing – original draft, Supervision, Software, Methodology, Formal analysis, Data curation, Conceptualization. **Dewi Stalpers:** Writing – review & editing, Validation, Conceptualization. **Catharina van Oostveen:** Writing – review & editing, Validation. **Maureen Rutten-Van Mölken:** Writing – review & editing, Supervision, Resources, Project administration, Methodology, Investigation, Funding acquisition, Conceptualization. **Lucas Goossens:** Writing – review & editing, Writing – original draft, Visualization, Validation, Supervision, Resources, Project administration, Methodology, Investigation, Funding acquisition, Formal analysis, Data curation, Conceptualization.

## Declaration of competing interest

The authors reports financial support was provided by Dutch Ministry of Health, Welfare and Sports.

## Appendix Supplementary materials

Image, application 1

Image, application 2

Image, application 3

Image, application 4

Image, application 5

Image, application 6

Image, application 7
