# Supplementary material for: Organisational interventions in nursing care: A scoping review and descriptive system to support comparison
Source: Int J Nurs Stud Adv. 2026 Jul 10;11:100626. doi: 10.1016/j.ijnsa.2026.100626 (PMC13383320; doi:10.1016/j.ijnsa.2026.100626)
Supplement: Supplementary file 1 [file mmc1.docx]

### Appendix A. Search strategy **Translation search query entries**

“nursing”, “differentiated nursing practices”, “organisation”, “nursing deployment”, “hospital” and “professional profile”

**Search strings**

| **Source** | **Search Term** | **Hits** |
| --- | --- | --- |
| google.nl | nursing role differentiation  nursing role differentiation pilot projects | 16900 (314)  102 |
| nfu.nl | differentiated nursing deployment  nursing role differentiation  professional nursing profiles | 0  0  0 |
| venvn.nl | differentiated nursing deployment  nursing role differentiation  professional nursing profiles (theme) | 3  44  9 |
| zorgvisie.nl | differentiated nursing deployment  nursing role differentiation | 7  30 |
| NIVEL | differentiated nursing deployment  nursing role differentiation  professional nursing profiles | 0  18  7 |
| nvz-kennisnet.nl | — | — |
| nursing.nl | differentiated nursing deployment  nursing role differentiation  professional nursing profiles | 13  172  203 |
| skipr.nl | differentiated nursing deployment  nursing role differentiation  professional nursing profiles | 4  22  30 |
| nvz-ziekenhuizen.nl | differentiated nursing deployment  nursing role differentiation  professional nursing profiles | 100  13  0 |
