# Supplementary material for: Organisational interventions in nursing care: A scoping review and descriptive system to support comparison
Source: Int J Nurs Stud Adv. 2026 Jul 10;11:100626. doi: 10.1016/j.ijnsa.2026.100626 (PMC13383320; doi:10.1016/j.ijnsa.2026.100626)
Supplement: Supplementary file 2 [file mmc2.docx]

### Appendix B. Coding system

| Indirect operational patient tasks **Text fragments examples (translated)** | **Elements** | **Dimensions** | **Themes** |
| --- | --- | --- | --- |
| *“For the required distinction within the pilot study, we use the terms ‘mbo educated nurses’ and ‘hbo educated nurses’, referring respectively to qualification levels NLQF 4 and NLQF 6.”* (R. de Vos & van Opstal, 2015, p. 5)  *“The development of a single educational profile by the 17 Universities of Applied Sciences is a positive initiative that has the potential to yield substantial benefits.”* (Jackson, 2015, p. 7)  *“In the Netherlands, three educational levels exist within the nursing domain: nurse specialists at level 7, bachelor‑educated nurses at level 6, and vocationally educated nurses at level 5.”* (A. de Vos et al., 2017, p. 18) | Education | Educational qualifications | Organisation of different types of nurses |
| *“There is no national definition of what constitutes a specialised nurse.”* (Pool et al., 2020, p. 9)  “The nursing profession should be divided into nurse specialists and general nurses.” (Hamel, 2019a) | Specialisation |  |  |
| *“The hospital hopes that nurses will seize the opportunity to further develop themselves, either within their team or beyond it. They are encouraged to do what is needed to improve the quality of care and to claim—and be given—the space to do so. This may lead to differences, but should not result in increased hierarchy. Nurses prefer to remain equal to one another.”* (Martini et al., 2021, p. 16) | Organisation of workforce (hierarchy) |  |  |
| *“A nurse with specialized dialysis training possesses many competencies that align with the level of a coordinating nurse, but only within the specific domain of dialysis care. A bachelor‑educated (HBO) nurse, however, holds these competencies across the full scale of nursing practice.”* (Gakes et al., 2018, p. 2) | Skills and talents | Competences |  |
| *“The EPAs clarify in which professional activities a student must become competent; which knowledge, skills, and attitudes are required; and which sources of information supervisors use to determine the appropriate level of supervision.”* (Pool et al., 2020, p. 11) | Qualifications |  |  |
| *“The pilot study focuses on the future positioning of the nursing domain. The emphasis is on distinguishing responsibilities, tasks, and roles.”* (R. de Vos & van Opstal, 2015, p. 4) | Responsibilities |  |  |
| *“In the pilot study, it became apparent that the motivation of coordinating nurses to implement role differentiation in practice varied. The motivation to step into the role appears to be an important factor in actively fulfilling the role.”* (Galama et al., 2019, p. 27) | Personal motivation and ambitions | Professional growth and disposition |  |
| *“The bachelor‑educated (hbo) nurse performs tasks independently, in accordance with standards, guidelines, and protocols, as well as with the instructions and orders of the supervisor and physician.”* (Peters & Alem, 2017, p. 12)  *“These documents clearly define the profiles of two types of nurses. … Essential to the distinction are the degree of coordination, initiative, autonomy, and the level of clinical skills.”* (Meijerink, 2017, p. 9) | Work independence |  |  |
| *“The personal development that healthcare professionals undergo must be documented and made transparent for patients and employers. We therefore propose the introduction of a personal portfolio. This would enable the appropriate deployment of individual healthcare professionals in practice.”* (Hamel, 2019b, p. 57) | Individual professional profile |  |  |
| *“…the committee’s report states that, for the role of coordinating nurse, attention should primarily be directed toward competencies, experience, and intrinsic motivation rather than solely the initial qualification.”* (Hamel, 2019b, p. 57) | Practices |  |  |
| *“The continuous cyclical process of clinical reasoning includes risk assessment, early detection, problem recognition, intervention, and monitoring. A well‑developed analytical capacity enables the bachelor‑educated (hbo) nurse to make appropriate decisions, even in situations where existing protocols provide insufficient guidance and it becomes necessary to integrate knowledge from different domains and to combine guidelines and protocols.”* (Geerts et al., 2015, p. 13) | Risks |  |  |
| *“None of the respondents believe that distinguishing levels of complexity in care within the specialist department offers opportunities to differentiate between various roles.”* (Peters, 2020a, p. 15)  *“I should note that from the outset we did not differentiate based on complexity. After all, complexity can change within a single day.”* (Hamel, 2019b, p. 55)  *“The instrument used to measure care complexity is highly suitable for determining which roles are needed at which moment.”* (van Lieshout et al., 2018, p. 2) | Complexity of care | Patient care | Organisation of different types of nursing work |
| *“In another emergency department, medical interns were deployed in supporting roles, handling logistical tasks and replenishing supplies. In an intensive care unit, special turning teams were introduced (to help reposition patients several times a day), laboratory teams were established, circulating nurses were added, and staff members were appointed to support the unit managers. In another intensive care unit, a buddy system was used, consisting mainly of anaesthesia staff and former intensive care nurses.”* (Peters, 2020b, p. 2)  *“There appears to be overlap in the tasks of the team leader, senior nurse, and coordinating nurse. This varies across pilot sites, depending on how the senior nurse role is defined. Whether, in addition to the coordinating nurse role, there is also a need for a senior nurse to lead projects or trajectories in the areas of quality improvement and/or education depends on the further design of the job structure.”* (Hillebrand et al., 2019, p. 10) | Organisation of patient care |  |  |
| *“The head nurse and the physician allocated the tasks that needed to be carried out that day among the nurses on duty. Later, nurses began to provide patient‑centred care. Nurses themselves became responsible for the patient’s overall care.”* (Martini et al., 2021, p. 9) | Patient oriented care |  |  |
| *… I was standing with a patient; she was crying, and I asked, ‘What can I do for you?’ … Then I felt the head nurse’s hand on my shoulder. She said, ‘This is not your task.’ That day I was on medication duty or whatever, so I was not allowed to get involved. (quote nurse)”* (Martini et al., 2021, p. 9)  *The inventory shows that all specialist departments mention differentiating work based on tasks.”* (Peters, 2020b, p. 1) | Task oriented care |  |  |
| *“In view of the objections to using complexity as a distinguishing characteristic between tasks suitable for vocational (mbo) nurses and bachelor (hbo) nurses—such as the difficulty of determining complexity in advance and the fact that it shifts within the dynamics of daily care practice—the job profiles instead make use of the unpredictability of the care situation.”* (Peters & Alem, 2017, p. 7) | Predictable care |  |  |
| *“They [coordinating nurses] are concerned with practice‑transcending responsibilities such as coaching and leadership; its distinguishing features lie particularly in analytical thinking and working.”* (Berkhout, 2019, p. 12) | Coaching | Indirect operational patient tasks |  |
| *“The introduction of nursing role differentiation confronted hospitals with a complex change process: the development of two distinct nursing function levels in daily practice. The first is the role of the nurse, an autonomous professional focused on direct patient care. The second is the role of the coordinating nurse, who—alongside direct patient care—has overarching coordinating responsibilities within the overall care process, as well as responsibilities in quality improvement, practice‑based research (including EBP), clinical reasoning, nursing leadership, and innovation.”* (Paardekooper, 2020, p. 7) | Coordination of nurses |  |  |
| *“Coordinating nurses developed themselves in the area of organisational tasks, such as coordinating patient flows and managing the team’s workload.”* (Schothorst-Van Roekel et al., 2017, p. 4) | Non patient related tasks |  |  |
| *“This requires nurses to critically examine care processes, using research where necessary. It also requires initiative to improve care, whether or not in collaboration with other professionals. In doing so, they can enhance patient care within the hospital. This would allow the nursing and medical domains to become equal to one another.”* (Martini et al., 2021, p. 13)  *“Previous international studies indicate that characteristics related to nursing professionalism—such as staffing levels and the composition of nursing teams—have an impact on the quality of care.”* (Stalpers, 2017, p. 10) | Quality of nursing care | Quality and safety |  |
| *“Within Excellent Care, one of the core principles is that sufficient staffing is required for the department to function effectively… Taking into account patient safety and the reduced availability of medical and nursing staff, the desired staffing ratio for the evening shift—when three nurses are present—is two bachelor‑educated nurses (hbo) and one vocationally educated nurse (mbo). During the night shift, the desired ratio is one bachelor‑educated nurse and one vocationally educated nurse.”* (R. de Vos & van Opstal, 2015, p. 27) | Safety of care |  |  |
| *“The profiles were developed from a theoretical perspective. In the pilot studies, we are testing them against practice. It is important to describe concretely what these profiles entail. What are the characteristics of the two levels? Under which conditions do both types of nurses perform best? The distinction becomes visible in the tasks, roles, and behaviours of the nurses, as well as in the requirements placed upon them.”*  (Deggens et al., 2018, p. 7) | Job classification (Duties, responsibilities, scope, and complexity of a position) | Positions and embedding | Terms of employment |
| *In addition to the professional profile, 22 areas of expertise have been described. These outline the additional competencies that nurses require in order to perform tasks within the respective domain.”* (Pool et al., 2020, p. 9) | Requirements |  |  |
| *“In the pilot projects, the positions of senior nurse, oncology nurse, and coordinating nurse existed alongside one another. The role of the senior nurse partially overlapped with that of the coordinating nurse.”* (Paardekooper, 2020, p. 12) | Position |  |  |
| *“V&VN [Dutch professional association for nurses] distinguishes three professions: care assistant, nurse, and nurse specialist. For each of these, professional and educational profiles have been developed.”* (Pool et al., 2020, p. 9)  *“There is considerable confusion surrounding the concepts of educational level, job level, professional profile, and job profile.”* (Gakes et al., 2018, p. 2)  *“Professional, job, and educational profiles form a triptych: each component influences the other two. The primary responsibility for these profiles lies respectively with the professional organisation, the healthcare institutions, and the universities of applied sciences.”* (Lambregts et al., 2015, p. 9) | Profession |  |  |
| *“Experimenting with differentiated practices of nursing at the hospital resulted in two distinct nursing roles. The role of the nurse: an all‑round professional focused on direct patient care. And the role of the coordinating nurse: all‑round in direct patient care, coordinator of care at the ward level, and responsible for quality improvement within the department. The coordinating nurse assumed a more overarching, analytical, and connecting role.”* (Schothorst-Van Roekel et al., 2017, p. 4) | Roles |  |  |
| *“Available resources—both financial and human—determine and limit the job mix.”* (van der Velde et al., 2019, p. 6) | Resources | Quantities and ratios |  |
| *“A group of nurses is given the responsibility to optimise the care for patients with a specific condition. This concerns the entire care pathway, meaning that other disciplines also join the expert team. In practice, this means that the nurses discuss bottlenecks in the care process, prioritise them, then analyse one selected bottleneck and subsequently develop an improvement plan.”* (Schroder & Donker, 2018, p. 127)  *“A Care Innovation Centre (ZIC) is a learning environment in which professionals in training, staff members, and educators work closely together.”* (Scheer & Westerbeek, 2021, p. 14) | Nursing team |  |  |
| *“Ward [anonymous] started the pilot with a staff mix of 50% vocationally educated nurses (mbo) and 50% bachelor‑educated nurses (hbo). This proved to be an ideal ratio for implementing differentiated practices of nursing.”* (Paardekooper, 2020, p. 12)  *“For the quality of professional practice, it is essential that employers create and offer a sufficient number of positions at both differentiated levels, and that an appropriate job mix is established.”* (Commissie Meurs, 2019, p. 16)  *“What is the ratio between interns and dual‑track students? And what is the ratio regarding the decline in mbo and hbo interns?”* (Jackson, 2015, p. 30) | Volume |  |  |
| *“According to several managers, the lack of further opportunities for salary progression may cause staff to refrain from taking on roles with greater responsibilities or additional tasks, such as the coordinating nurse role. … One of the managers expressed the hope that national agreements will be established on this matter, as she expects that otherwise nurses will leave for hospitals that do offer higher remuneration for specialised nurses.”* (Peters, 2020a, p. 6)  *On the other hand, employees must be supported as much as possible in pursuing their ambitions, for which assessments can be important instruments. The design of training pathways, clear expectation management regarding their nature, appropriate compensation and flexibility, as well as sufficient guidance and protected time (particularly in light of the crucial work–life balance), are the key preconditions.”* (van der Velde et al., 2019, p. 11)  *There is sufficient room for additional training (expressed in both time and financial resources, including backfill costs).”* (Secchi, 2017, p. 3) | *Conditions of job (salary, formal work, authority)* | *Recognition and rewards* |  |

Berkhout, C. (2019). Nieuw: De Regieverpleegkundige. *Leeuwarder Courant*, 12.

Commissie Meurs. (2019). Een overgangsregeling in het kader van Big II register. *De verpleegkundig specialist*, 23.

de Vos, A., Vilrokx, C., & Olsthoorn, E. (2017). Verpleegkundigen maken het verschil: Programma voor functiedifferentiatie mbo-hbo in Amphia. *Nederlands Tijdschrift voor Evidence Based Practice*, 18-24.

de Vos, R., & van Opstal, D. (2015). *Excelleren op beide niveaus* (p. 60). Amphia Ziekenhuis.

Deggens, J., Meijwaard, E., Samuels, A., & Schotman, I. (2018). *Handreiking proeftuinen VIP* (p. 23). Nederlandse Federatie van Universitair Medische Centra.

Gakes, A., Velderman, L., van Zalingen, V., de Bruin, L., van den Brink, T., Hermann, L., & van den Essen, M. (2018). *Advies functiedifferentiatie op de dialyseafdeling* (pp. 1-6). V&VN.

Galama, J., Duismann, J., Hopman, H., & Naber, A. (2019). *Proeftuinen Amsterdam UMC* (p. 92). Amsterdam UMC.

Geerts, I., van ’t Zelfde, P., Terpstra, D., Van den Berg, A., Van Mierlo, C., Zijlstra, H., Landman, J., Schuurmans, M., & Kempff, M. (2015). *Toekomstbestendige beroepen in de verpleging en verzorging* (p. 69). Stuurgroep Beroepsprofielen en de overgangsregeling.

Hamel, M. (2019a). De lange weg naar splitsing van functies. *Nursing*, 18-23.

Hamel, M. (2019b). Functie-differentiatie: Ziekenhuizen zetten door. *Nursing*, 54-59.

Hillebrand, M., Kramer, D., Smilde, D., Weijwaard, E., Busato, C., & Schotman, I. (2019). *Verpleegkundige Innovatie & Positionering. Bevindingen en praktijkervaringen uit de proeftuinen van de umc ’s.* (p. 24). Nederlandse Federatie van Universitair Medische Centra.

Jackson, W. (2015). *Leren in het ziekenhuis. Een verkenning naar leren in de praktijk van hbo-studenten verpleegkunde*. ETZ. http://www.loov2020.nl/wp-content/uploads/2015/05/Leren-in-het-ziekenhuis.pdf

Lambregts, J., Grotendorst, A., & Van Merwijk, C. (2015). Bachelor Nursing 2020. In *Landelijk overleg opleidingen verpleegkunde* (pp. 1-66). Landelijk Overleg Opleidingen Verpleegkunde.

Martini, K. D., Schalkwijk, H., Smit, G. A. C., & Lalleman, P. (2021). *De Verpleegkundige van Morgen: Een leergeschiedenis over verpleegkundig werk en de gedifferentieerde inzet van verpleegkundigen in Rijnstate* (p. 27).

Meijerink, M. (2017). De juiste verhouding: Een vernieuwend verpleegkundig beroepsprofiel. *I.O. VUmc Maganzine voor onderwijs en opleiden*, 8-11.

Paardekooper, M. (2020). *Project Proeftuinen Toekomstbestendige Verpleegkundigen* (pp. 1-36). HMC - Haaglanden Medisch Centrum.

Peters, F. (2020a). *De gedifferentieerde inzet van verpleegkundigen op specialistische afdelingen* (p. 53). KBA.

Peters, F. (2020b). *De uitbraak van de coronacrisis en de inzet van verpleegkundigen op gespecialiseerde afdelingen* (pp. 1-8). KBA.

Peters, F., & Alem, D. (2017). *Functiedifferentiatie mbo- en hbo opgeleide verpleegkundigen: Functieprofielen en leidraad voor de invoering* (p. 52). Nederlandse Vereniging van Ziekenhuizen.

Pool, I. A., Ten Cate, O., Deggens, J., Hofstra, S., Van der Horst, M., Overeem, E., & Van Zandvoort, M. (2020). *Contouren van het nieuwe CZO-opleidingsstelsel*. Stuurgroep CZO Flex Level.

Scheer, S., & Westerbeek, A. (2021). *Slimmer werken, samen werken: Vernieuwend werken in tijden van crisis en krapte* (p. 44). Nederlandse Vereniging van Ziekenhuizen.

Schothorst-Van Roekel, J., Weggelaar-Jansen, A., & Wallenburg, I. (2017). *Verpleegkundige functiedifferentiatie in het Reinier de Graaf ziekenhuis. Tussenrapportage van een jaar experimenteren in proeftuinen.* (p. 27). Erasmus University Rotterdam. https://www.eur.nl/sites/corporate/files/2019-02/Schothorst_verpleegkundige_functiedifferentiatie_171013.pdf

Schroder, P., & Donker, M. (2018). Verpleegkundige functiedifferentiatie: Een uitdagend middel om de zorg te optimaliseren. *Cordiaal*, (5). https://www.nvhvv.nl/wp-content/uploads/2018/10/Cordiaal_2018-4_LR.pdf

Secchi, R. (2017). *Proeftuin MBO-HBO verpleegkundigen MUMC+*. MUMC+.

Stalpers, D. (2017, mei). Functiedifferentiatie mbo- en hbo-opegeleide verpleegkundigen. *Loupe*, (2), 2.

van der Velde, F., Aalbers, W., Bloemendaal, I., Detaille, S., & Verbruggen, W. (2019). Arbeidsmarktonderzoek toekomstbestendige beroepen in de verpleging en verzorging. In *Ministerie van Volksgezondheid, Welzijn en Sport* (pp. 0-100).

van Lieshout, J., Boerboom-Koehorst, A., van Nooten, A., van Hulst, J., De La Haye, J., van Loveren, M., Reynders-Stol, B., van Mackelenbergh, S., Leenders, C., Kouwen, A., & Ter Sluysen, J. (2018). *De verpleegkundige van de toekomst: Door scherpere rolverdeling betere zorg* (p. 105). Radboudumc.
