# Supplementary material for: Organisational interventions in nursing care: A scoping review and descriptive system to support comparison
Source: Int J Nurs Stud Adv. 2026 Jul 10;11:100626. doi: 10.1016/j.ijnsa.2026.100626 (PMC13383320; doi:10.1016/j.ijnsa.2026.100626)
Supplement: Supplementary file 3 [file mmc3.docx]

### Appendix C. Checklist descriptive system

#### Theme 1: Organising different types of nurses

##### Educational qualifications

**CURRENT organisation of nurses**

1. Are educational qualifications relevant when describing the CURRENT organisation of nurses?

No

Yes

1. Select all relevant topics describing educational qualifications within the CURRENT organisation of nurses

No relevant topics

Vocational degrees

Bachelors’ degrees

Masters’ degrees

Nursing students

Training

Post-educational specialisation

Other, please specify: …………….

**INTERVENTION of organising nurses**

1. Has there been an introduction or change in educational qualifications related to the INTERVENTION of organising nurses?

No

Yes

1. Select all relevant topics describing educational qualifications regarding the INTERVENTION of organising nurses

No relevant topics

Hierarchy

Legislation

Educational requirements

Accreditation

Training

Other, please specify: …………….

##### Competences

**CURRENT organisation of nurses**

1. Are competences relevant when describing the CURRENT organisation of nurses?

No

Yes

1. Select all relevant topics describing competences within the CURRENT organisation of nurses

No relevant topics

Current competence method or model

Canadian Medical Education Directions for Specialists (CanMEDS)

Entrustable Professional Activities (EPA)

Other, please specify: …………….

**INTERVENTION of organising nurses**

1. Has there been an introduction or change in competences related to the INTERVENTION of organising nurses?

No

Yes

1. Select all relevant topics describing competences regarding the INTERVENTION of organising nurses

No relevant topics

New competence method or model

Professional insights

Personal attitudes

Professional capabilities

Skill sets

Other, please specify: …………….

##### Professional growth and disposition

**CURRENT organisation of nurses**

1. Is professional growth and disposition relevant when describing the CURRENT organisation of nurses?

No

Yes

1. Select all relevant topics describing professional growth and disposition within the CURRENT organisation of nurses

No relevant topics

Autonomy

Professional experiences

Intrinsic motivation

Other, please specify: …………….

**INTERVENTION of organising nurses**

1. Has there been an introduction or change in professional growth and disposition related to the INTERVENTION of organising nurses?

No

Yes

1. Select all relevant topics describing professional growth and disposition regarding the INTERVENTION of organising nurses

No relevant topics

Individual personal portfolio

Personal career ambitions

Other, please specify: …………….

#### Theme 2: Organising different types of nursing work

##### Patient care

**CURRENT organisation of nursing work**

1. Is patient care relevant when describing the CURRENT organisation of nursing work?

No

Yes

1. Select all relevant topics describing patient care within the CURRENT organisation of nursing work

No relevant topics

Team-based nursing care organisation

Task-based nursing care organisation

Tasks

Duties

Responsibilities

Other, please specify: …………….

**INTERVENTION of organising nursing work**

1. Has there been an introduction or change in patient care related to the INTERVENTION of organising nursing work?

No

Yes

1. Select all relevant topics describing patient care regarding the INTERVENTION of organising nursing work

No relevant topics

Complexity of care

Predictability of care

Specialised patient care

Other, please specify: …………….

##### Indirect operational patient tasks

**CURRENT organisation of nursing work**

1. Is indirect operational patient tasks relevant when describing the CURRENT organisation of nursing work?

No

Yes

1. Select all relevant topics describing indirect operational patient tasks within the CURRENT organisation of nursing work

No relevant topics

Coordination of care

Coaching of team members

Management of care

Other, please specify: …………….

**INTERVENTION of organising nursing work**

1. Has there been an introduction or change in indirect operational patient tasks related to the INTERVENTION of organising nursing work?

No

Yes

1. Select all relevant topics describing indirect operational patient tasks regarding the INTERVENTION of organising nursing work

No relevant topics

Professionalisation of the work environment

Other, please specify: …………….

##### Quality and research

**CURRENT organisation of nursing work**

1. Is quality and research relevant when describing the CURRENT organisation of nursing work?

No

Yes

1. Select all relevant topics describing quality and research within the CURRENT organisation of nursing work

No relevant topics

Safety and regulation

Protocols

Guidelines

Other, please specify: …………….

**INTERVENTION of organising nursing work**

1. Has there been an introduction or change in quality and research related to the INTERVENTION of organising nursing work?

No

Yes

1. Select all relevant topics describing quality and research regarding the INTERVENTION of organising nursing work

No relevant topics

New regulations

Changing protocols or guidelines

Participation in committees

Reflection and feedback

Other, please specify: …………….

#### Theme 3: Terms of employment

##### Positions and embedding

**CURRENT terms of employment**

1. Are positions and embedding relevant when describing the CURRENT terms employment?

No

Yes

1. Select all relevant topics describing positions and embedding within the CURRENT terms of employment

No relevant topics

Professional profile

Educational profile

Nursing job profile

Nursing roles

Nursing position

Other, please specify: …………….

**INTERVENTION in terms of employment**

1. Has there been an introduction or change in positions and embedding related to the INTERVENTION of organising nursing work?

No

Yes

1. Select all relevant topics describing positions and embedding regarding the INTERVENTION of organising nursing work

No relevant topics

Professional requirements

Professional behaviours

Responsibilities

Tasks

New or changing profile

New or changing role

Other, please specify: …………….

##### Quantity and ratios

**CURRENT terms of employment**

1. Are quantity and ratios relevant when describing the CURRENT terms employment?

No

Yes

1. Select all relevant topics describing quantity and ratios within the CURRENT terms of employment

No relevant topics

Skill mix

Nurse-patient ratio

Shift patterns

Mix of positions

Fulltime / parttime employment

Other, please specify: …………….

**INTERVENTION in terms of employment**

1. Has there been an introduction or change in quantity and ratios related to the INTERVENTION of organising nursing work?

No

Yes

1. Select all relevant topics describing quantity and ratios regarding the INTERVENTION of organising nursing work

No relevant topics

Change in skill mix

New nurse-patient ratio

Changing shift patterns

New mix of positions

Change in fulltime / parttime employment

Other, please specify: …………….

##### Recognition and rewards

**CURRENT terms of employment**

1. Are recognition and reward relevant when describing the CURRENT terms employment?

No

Yes

1. Select all relevant topics describing recognition and reward within the CURRENT terms of employment

No relevant topics

Monetary reward

Non-monetary reward

Other, please specify: …………….

**INTERVENTION in terms of employment**

1. Has there been an introduction or change in recognition and reward related to the INTERVENTION of organising nursing work?

No

Yes

1. Select all relevant topics describing recognition and reward regarding the INTERVENTION of organising nursing work

No relevant topics

Salary

Student compensation

Additional educational opportunities

Time for research and development

Opportunities for career advancement

Other, please specify: …………….

#### Context

##### Patient related contextual factors

| Please give brief description of relevant patient-related contextual factors in ca. 50 words. |
| --- |
| *Examples of patient related contextual factors are: disease, treatment, specialised care aspects or patient characteristics.* |

##### Organisational contextual factors

| Please give brief description of relevant organisational contextual factors in ca. 50 words. |
| --- |
| *Examples of organisational contextual factors are: work processes, professional conduct framework, organogram, design of the work floor, geographical factors* |

##### Country related context

| Please give brief description of relevant country-related contextual factors in ca. 50 words. |
| --- |
| *Examples of country contextual factors are: labour market, professional conduct framework, culture, nurse-population ratios, access of care, political factors, health care system, legislative factors or economic factors (i.e. payer system, national health care expenditure).* |

| Please give a coherent overview of the organisational intervention of nursing care using all relevant dimensions in ca. 300-600 words. |
| --- |
|  |
