# Supplementary figures and images for: Organisational interventions in nursing care: A scoping review and descriptive system to support comparison
Source: Int J Nurs Stud Adv. 2026 Jul 10;11:100626. doi: 10.1016/j.ijnsa.2026.100626 (PMC13383320; doi:10.1016/j.ijnsa.2026.100626)

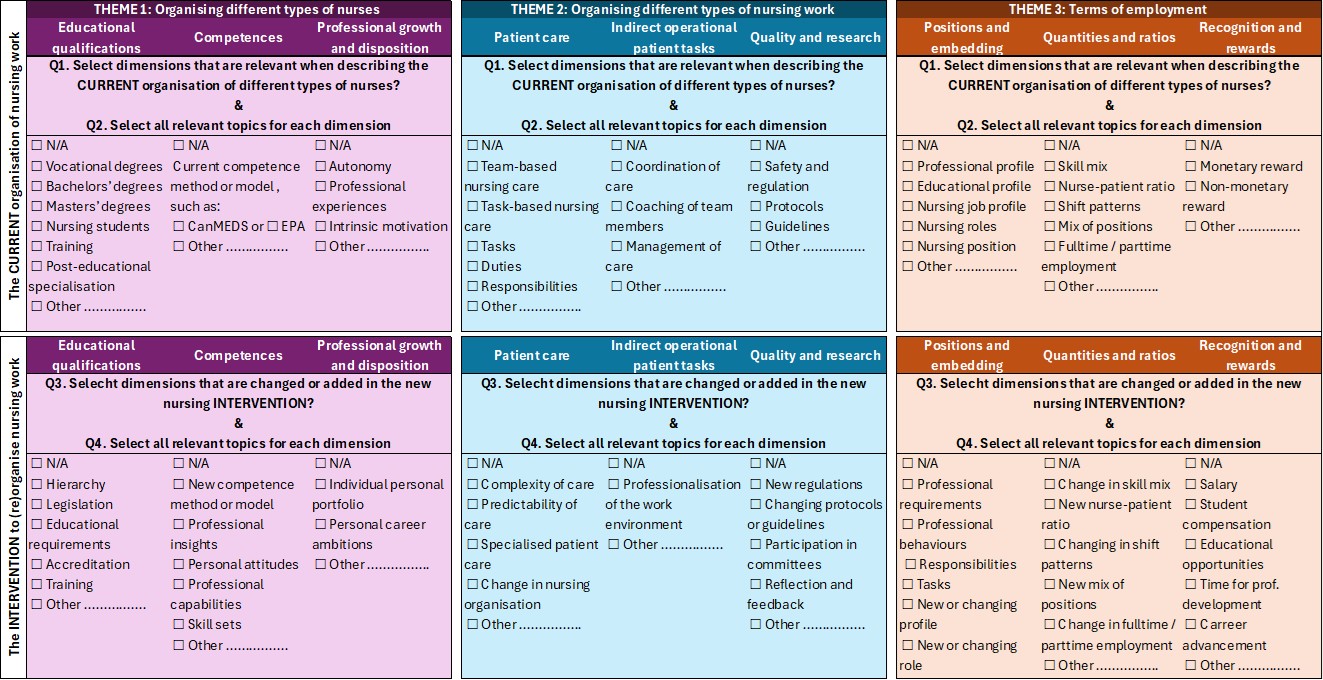
Appendix D. Matrix descriptive system

Supplement: Supplementary file 4 [file mmc4.docx]
