# Supplementary material for: Organisational interventions in nursing care: A scoping review and descriptive system to support comparison
Source: Int J Nurs Stud Adv. 2026 Jul 10;11:100626. doi: 10.1016/j.ijnsa.2026.100626 (PMC13383320; doi:10.1016/j.ijnsa.2026.100626)
Supplement: Supplementary file 5 [file mmc5.docx]

### Appendix E. Overview grey literature

| **Author** | **Year** | **Title** | **Type of document** | **Aims/objective** | **Focus of report** | **Relevant findings** |
| --- | --- | --- | --- | --- | --- | --- |
| Paardekoper | 2020 | Pilot Project Future-Proof Nurses (Project Proeftuinen Toekomstbestendige Verpleegkundigen) | Institute report | To evaluate the final outcomes and lessons of experimenting with new and innovative ways to organising the nursing profession within a hospital | Differentiated practices of nursing, job profiles, nursing organisation | The report described the development and experimentation with nursing organisation by differentiating the nursing workforce in two distinct groups based on **level of education**. Nurses with a bachelors' degree were responsible for additional tasks beyond individual patient care such as **coordination of patient** care within the whole unit, integrating **quality of care**, researching **Evidence Based Practices**, **clinical reasoning**, nursing **leadership** and **innovation of care**. Furthermore, different **skill mix** models were introduced based on different **contexts** of nursing units to see which **function mix** would be optimal. Relevant **contextual factors** within a nursing ward included the **demand of care**, **organisation of nursing work**, previous **team mix** and **development stages** within a nursing unit. |
| de Vos, R. & van Opstal, D. | 2015 | Excelling at Both Levels: Pilot Project on Nursing Job Profiles for MBO and HBO (Excelleren op beide niveaus: Proeftuin verpleegkundige functieprofielen mbo hbo) | Institute report | To describe the process and recommendations of the experiments with new and innovative ways to organising the nursing profession within a hospital | Nursing job profiles | The report described the organisation and implementation of new **nursing profiles** developed for **bachelor nurses** and vocational **nurses** in addition to the existing profiles for **nurse practitioners** and nurses with **additional specialised training**. The profiles defined each nursing position based on **educational level**, **professional requirements**, **work activities**, **valuation** and the **positioning** within the organisation. |
| Peters & Alem | 2017 | Differentiated Practices of Nursing between Vocational and Bachelor Educated Nurses (Functiedifferentiatie mbo- en hbo-opgeleide verpleegkundigen) | Institute report | To clarify new professional nursing profiles created for general hospitals and present a guideline how to implement differentiated practices of nursing within general hospitals | Differentiated practices of nursing, job profiles | The report described two **professional nursing profiles** for nurses with a bachelors' degree and a vocational degree. Both profiles display the fundamentals needed based on patient **care**, **expertise** and **knowledge**, **autonomy** and **responsibilities**, and **valuation** of the degree. Further elaboration describes specific **tasks** and **requirements** as presented by Lambregts et al (2015) in the educational profiles. The report specifically displayed the difference differentiating nursing practised based on **complexity of care** and **unpredictability of care**. This report preferred the latter. |
| Jackson, W. | 2015 | Learning in the Hospital (Leren in het ziekenhuis) | Institute report | To conduct a further exploration of the theme internships in a professional practice | Nursing internships | This report explored the topic of **internships** within a professional hospital setting. The report focussed on three main topics: how to optimise the capacity of internships within the hospital, the factors needed to optimise the quality of internships within the hospital, and how to **embed the educational profile** within the hospital. |
| Secchi, R | 2017 | Pilot Project Vocational and Bachelor Nurses at MUMC+ (Proeftuin MBO-HBO verpleegkundigen MUMC+) | Institute report | To describe the process of experimenting and implementing innovative concepts of job profiles within an academic hospital | Nursing job profiles | Two professional nursing **profiles** for nurses with a **bachelors' degree** and nurses with a **vocational degree** were introduced in 2016. These profiles were used to develop a new concept of **nursing job profiles** within teaching hospitals. The new nursing job profiles are not viewed as an established framework but served as the basis to experiment with the organisation of the nursing workforce in several nursing units. The experiments are used to create distinct **roles**, **tasks**, and **responsibilities** for each **level of education** within the context of the nursing units. Key concepts in the development: advancing **quality of care**, creating optimal **function mix**, and developing **leadership**, **autonomy**, **professionalism**, **control** and **responsibility** within the nursing workforce. |
| Deggens et al. | 2018 | Guide for Pilot Projects VIP (Handreiking proeftuinen VIP) | Institute report | A guideline to advice teaching hospitals how to redefine nursing job profiles and experiment with function mix within academic hospitals | Differentiated practices of nursing, job profiles, function mix | A guideline with four steps how to prepare experiments with reorganising the nursing workforce within teaching hospitals: (1) recalibrate the vision of nursing care, (2) delineate specific **nursing tasks** and **competences**, (3) plan the experiment based on goals, structure, needs and time, and (4) communicate the plans within the whole organisation. Specific experiments and plans depend on the **context** of each hospital and/or nursing unit. **Diversity** is encouraged by assessing the **current state** of each nursing workforce to provide **context** specific outcomes on optimal use of **function mix** within a team. |
| Hillebrand et al. | 2019 | Findings and Practical Experiences from the Academic Hospitals' Pilot Projects (Bevindingen en praktijkervaringen uit de proeftuinen van de umc’s) | Institute report | To evaluate the first outcomes and findings of redefining new nursing job profiles and experiments with function mix within academic hospitals | Differentiated practices of nursing, job profiles, function mix | Relevant outcome showed that function mix is extremely **context** specific and depends on various **factors**. Factors identified in this report are: **nursing care methods**, current **composition** **of nursing teams**, **developmental phase** of nursing team, the current state of the **nursing labour market**, current and future **demand of care**, the **design of the whole nursing workforce** in a hospital and specific care characteristics of a nursing unit such as; the **work process**, **specialised care** and predictability **or care**. |
| Van Lieshout | 2018 | The Nurse of the Future: Better Care through Sharper Role Delineation (De verpleegkundige van de toekomst: door scherpere rolverdeling betere zorg) | Institute report | To evaluate the outcomes and recommendations of implementing new nursing organisation structures within a academic hospital | Differentiated practices of nursing, job profiles | Relevant outcomes: differentiating nurses based on **educational level** is effective to increase attention to patient care, **complexity of care** is a good tool to differentiate nursing roles, well defined and complete distinct **nursing roles** increase team spirit, differentiated **nursing roles** increases **professionalism** in the nursing workforce and is in line with **patient needs**, **valuation** of differentiated practices of nursing needs more consideration. |
| Geerts, I., van ’t Zelfde, P., Terpstra, D., van den Berg, A., van Mierlo, C., Zijlstra, H., Landman, J., Schuurmans, M., & Kempff, M. | 2015 | Future-Proof Professions in Nursing and Care (Toekomstbestendige beroepen in de verpleging en verzorging) | Institute report | To advice the government for legislative amendments regarding professional nursing profiles | Professional nursing profiles | The report introduces three professional nursing profiles: the bachelor nurse, the vocational nurse and the nursing aid. All profiles are outlined based on fields of **expertise**, **competences**, and the **authority** by law to perform procedures protected by legislation. **Expertise** is described as professional **autonomy**, where a professional is able to independently shape their role based on their **knowledge** and **skills**. Competences are based on **knowledge**, **skills** and **attitudes** devided in seven distinct roles: health care provider, communicator, collaborator, reflective EBP professional, health promotor, organiser, and promotor of professionalism and quality. |
| Galama et al | 2019 | Evaluation and Advisory Report on New Nursing Professional Profiles (Evaluatie- en adviesrapport Nieuwe verpleegkundige beroepsprofielen) | Institute report | To evaluate the outcomes and recommendations of experimenting with differentiated practices of nurses, nursing job profiles, and nursing function mix within a academic hospital | Differentiated practices of nursing, nursing job profiles, function mix | The report evaluated the outcomes and recommendations after experimenting with two new **nursing job profiles**. Prior to the experiment, nurses with two different **educational profiles** (bachelors' and vocational degree) were working within the same **job profile**, regular nurse. Two new job profiles were introduced which differentiated two **functions** based on both **educational degrees**. |
| Commissie Meurs | 2019 | Nursing at the Right Level: A Transitional Arrangement under the BIG II Register (Verpleegkunde op niveau: een overgangsregeling in het kader van BIG II register) | Legislative proposal | To update and reform the nursing profession by introducing a new nursing role embedded in the Dutch health care professional legislation | Differentiated practices of nursing, educational levels, health care professional legislation | This report proposed a method of **differentiated practices of nursing** by introducing a **new nursing role**: the nurse coordinator. This new role is based on the educational **background**, field of **expertise** and acquired **competences** of each individual nurse. The proposal aimed to embed this new nursing role within the Dutch health care professional legislative system. |
| Schroder | 2018 | Differentiated Practices of Nursing (Verpleegkundige functiedifferentiatie) | Professional literature | To discuss the process of implementing differentiated practices of nursing within a Dutch hospital | Differentiated practices of nursing, nursing work, nursing organisation | The article discussed the process of developing and implementing **differentiated practices of nursing** within a Dutch hospital. The process described differentiation based on various **nursing competences** based on different **educational levels** and **job profiles**. Implementing these **two distinguished profiles** proved to be complex and implementation is difficult. |
| Hamel | 2019 | Differentiated Practices of Nursing: The Long Division (Functiedifferentiatie: de lange splitsing) | Professional literature | The aim of the article was to provide an timeline of differentiated practices of nursing over the last 100 years within the Dutch nursing landscape | Differentiated practices of nursing, timeline | The article describes the timeline of **differentiated practices of nursing** within the Dutch nursing landscape. The article describes the transition in **educational differences**, legislation and the impact on the Dutch nursing profession. Furthermore, it describes challenges and discourse among the national actors involved in the transition. |
| Hamel | 2019 | Differentiated Practices of Nursing: Hospitals push ahead (Functiedifferentiatie: ziekenhuizen zetten door) | Professional literature | Discussing the development of differentiated practices of nursing between nurses with a vocational degree and a bachelors' degree | Differentiated practices of nursing | The article discussed the attempts to develop a **new nursing position**: the directing nurse. This position was introduced to differentiate nurses based on **level of education** and delivering different types of **complexity of care**. However, the article described a more layered composition of additional relevant topics in organising differentiated practices of nursing: **advanced trainings/specialisations**, **competences**, **nursing** **leadership**, how to manage **hierarchy**, **clinical** **reasoning**, new nursing **roles**, **valuation** of nurses, **fluctuations in complexity of care**, **quality** and **safety** of care, and **professional experience** and **intrinsic professional motivation**. |
| Meijerink, A. | 2017 | The Right Balance (De juiste verhouding) | Professional literature | To discuss the developments regarding new nursing profiles based on differentiated practices of nursing | Differentiated practices of nursing, job profiles, nursing education | The article discussed the development of a **new nursing profile**, the nurse coordinator, and the possibilities of implementing this new **profile** within the hospital. The new profile is based on specific **competences**. These competences are in line with two different **nursing degrees**: the vocational nurse and the bachelor nurse. To implement this **new profile** the **educational programs** should align with the **job profiles** offered within the hospital. |
| Stalpers | 2017 | Differentiated Practices of Nursing between Vocational and Bachelor Educated Nurses (Functiedifferentiatie mbo- en hbo-opgeleide verpleegkundigen) | Professional literature | To describe the development of differentiated practices of nursing within the Dutch nursing landscape and within a specific Dutch hospital | Differentiated practices of nursing | The article described three types of **nursing profiles**: the **professional profile**, the **educational profile** and the **job profile**. These three profiles are all connected and in line with one another. |
| Berkhout | 2019 | The Nurse Coordinator (De regieverpleegkundige) | Professional literature | To describe how four hospitals experimented with the implementation of a new nursing role: the nurse coordinator | Differentiated practices of nursing, experiments, nursing role, educational differentiation | The article described the implementation of a new nursing role: the nurse coordinator. The current nursing profiles in all hospitals does **not differentiate** **between educational backgrounds**. The new **nursing role** does not change direct patient care, but focuses on **care transcending tasks** such as **coaching**, **leadership**, and **reflection**. The new role is implemented in teams with pilot cases and aims to focus on **quality of care** and **teamwork**. |
| de Vos, Vilrokx & Olsthoorn | 2017 | Nurses Make the Difference (Verpleegkundigen maken het verschil) | Professional literature | To describe the layout of a new program of differentiated practices of nursing | Differentiated practices of nursing, educational differentiation, | The article outlined a program framework set up to **differentiate practices of nursing** for nurses with **vocational and bachelors’ degrees** in a local hospital. The aim of the program was to enhance **quality**, **safety** and affordability of patient care. The objectives of the program were **role differentiation**, **empowerment** of nurses and **professional development**. The program was implemented from a bottom-up approach, engaging all layers of the organisation and to create a learning environment. |
| Lambregts, J., Grotendorst, A., van Merwijk, C. | 2015 | Bachelor Nursing 2020: A Future-Proof Educational Profile 4.0 (Bachelor Nursing 2020: Een toekomstbestendig opleidingsprofiel 4.0) | Policy report | To contextualise the educational framework for the Bachelor of Nursing | Educational framework | The report contextualised the **training profile** for the Bachelor of Nursing. Three types of **nursing profiles** are introduced: the **professional profile**, the **job profile,** and lastly the **training profile**. These profiles are viewed as extensions of each other with each component within a profile influencing the other profiles. The training profile is outlined based on key concepts and competences of **knowledge**, **skills** and **attitudes** needed to obtain a bachelors' degree. Nursing competences are divided in seven distinct roles: health care provider, communicator, collaborator, reflective EBP professional, health promotor, organiser, and promotor of professionalism and quality. |
| Scheer & Westerbeek | 2021 | Working Smarter, Working Together (Slimmer werken, samen werken) | Program report | To describe various themes and examples of developments to balance the nursing workforce in face of the COVID-19 outbreak | Nursing workforce developments, COVID-19 | Three themes were presented: shortage in the nursing workforce, optimal arrangements and employment of health care professionals, and changes in health care due to technology developments. Relevant findings showed that the implementation of a new **learning centre** for specialised nurses in training in an Emergency Room where trained nurses were deployed as qualified professionals. This created an increase in **training capacity**, coaching **skills**, and an innovative **learning environment** for the nursing unit. |
| Peters | 2020 | The Outbreak of the COVID Crisis and the Deployment of Nurses in Specialist Departments (De uitbraak van de coronacrisis en de inzet van verpleegkundigen op specialistische afdelingen) | Research report | To assess and survey how specialised nursing departments deploy differentiated practices of nursing during and post the COVID pandemic | Differentiated practices of nursing, nursing work, nursing organisation, specialised nursing departments | The report discussed the outcomes of a large survey conducted within twelve specialised nursing departments. This survey was conducted to assess the developments with **differentiated practices of nursing** forced by the challenges of the COVID pandemic. The main development within all departments was the **substitution of care activities** and with that the (re)introduction of **task-based nursing organisation**. This development was mainly forced due to the mayor shortages of nurses combined with the high influx of - mostly complex - patient counts. |
| Martini, K.D., Schalkwijk, H., Smid, G.A.C., Lalleman, P.C.B. | 2021 | The Nurse of Tomorrow: A Learning History of Nursing Work and Differentiated Deployment in Rijnstate (De Verpleegkundige van Morgen: Een leergeschiedenis over verpleegkundig werk en de gedifferentieerde inzet van verpleegkundigen in Rijnstate) | Research report | A learning history of the nursing workforce development within a hospital | Differentiated practices of nursing, nursing work, nursing workforce and nurses organisational influence | The report discussed the major developments within the nursing workforce over time. Important themes were: organisational development from **task-oriented nursing care** to **patient-oriented nursing care**, the friction and importance of **practical experience** and/or **level of education**, the friction between d**ifferentiation of nursing** and **teamwork**, the transition of **delegating** nursing tasks to **supporting staff**, introducing and experimenting with different levels of nursing **qualifications**, introducing new nursing **positions** and/or **roles** based on **competences**, **invisible nursing work** and/or **nursing work not directly bound to the patient**, nursing **authority** and **autonomy** of organising care, nursing teams with different **backgrounds**, level of **education**, **advanced training/specialisation**, **preferences**, **expertise/skills,** and **competences**. |
| van der Veld, F., Aalbers, W., Bloemendaal, I., Detaille, S., & Verbruggen, W. | 2019 | Labour Market Research on Future-Proof Professions in Nursing and Care (Arbeidsmarktonderzoek toekomstbestendige beroepen in de verpleging en verzorging) | Research report | To assess professional nursing profiles and the future of the nursing labour market | Professional nursing profiles | The report discussed three distinct **nursing profiles**: **the bachelor nurse**, the **vocational nurse** and **the nursing aid**. The nursing profiles are assessed based on three perspectives: **care**, **people** and **resources**. Each perspective is a factor in determining future **function mix** of nurses and displayed as a scenario with different **mixes of people**, shortages and surplus of **resources** and **people**, and **costs** of **education** and **salary**. The **care perspective** showed a mix of nurses based on care demands and care organisation. The people perspective displayed nurse mixes based on **educational degrees**, **competences**, **personal ambition,** **career development** and **training** potential. The last perspective, **resources**, described the limitations of **team mixes** due to **budgetary restraints** and **shortages of personnel**. |
| Peters | 2020 | The Differentiated Deployment of Nurses in Specialist Departments (De gedifferentieerde inzet van verpleegkundigen op specialistische afdelingen) | Research report | To assess how specialised nursing units, such as emergency departments and intensive care units, can characterise different levels of nursing positions and specialisations | Differentiated practices of nursing within specialised nursing units | The reports described two concepts of differentiated practices: the **differentiation of nursing tasks** and the **differentiation of nursing positions**. **Differentiation of nursing** tasks was defined as nurses within the same **position** responsible for a (partly) different set of **tasks**. Observed examples were different **tasks** for nurses with different **roles**, **focus of area** or **specialisation**. **Differentiation of nursing positions** was defined as different positions of nursing with a unit. Observed examples were regular nurses, specialised nurses, directing nurses, nurses with a master’s degree and aid workers. Reasons identified to further develop differentiation in specialised nursing units were organising **complexity of care**, utilising **individual competences** and **educational skills**, and creating **development opportunities** and **career prospectives**. |
| Van Schothorst et al | 2019 | Nurses in Control: Final Report on Two Years of Experimenting with Differentiated Practices of Nursing in Reinier de Graaf Hospital (Verpleegkundigen in regie: Eindrapportage van twee jaar experimenteren met functiedifferentiatie in het Reinier de Graaf Gasthuis) | Research report | To assess the consequences of experimenting with differentiated practices of nursing for nurses, other health care professionals, managers, de patient and the organisation of work processes within a general hospital | Differentiated practices of nursing | The hospital experimented with two different nursing roles, the **directing nurse** and a **regular nurse**. These roles were used to formalised new **nursing functions** within the hospital. Distinction was made based on **educational levels**, **complexity of care**, and specific directing **tasks** such as **coordination** and **quality** **of care**. The directing nurse was expected to surpass general nursing care by implementing a more **analytical** and **connecting** role. Relevant outcomes showed that differentiating nursing practices is difficult based on complexity of care, the directing nurse tasks were mostly shaped as **indirect organisation of care**, new and old nursing **functions overlap** in tasks, differentiating nurses based on **educational levels** should factor in recently graduated nurses, and a **function mix** of 30% directing nurse with 70% regular nurses was needed to implement new work structures. |
| Gakes et al. | 2018 | Advisory Report on Diferentiated Practices of Nursing in the Dialysis Department (Advies functiedifferentiatie op de dialyseafdeling) | Working group report | To research and advice which competences - assessing job profiles and expertise areas - are needed within the nursing dialysis workforce to care for patients within the nephrology domain. Furthermore, the report assesses what optimal function mix is required between vocational and bachelor nurses based on competences. | Differentiated practices of nursing, educational levels, nursing organisation | The report is based on the notion that the nursing workforce can be distinguished based on t**wo educational levels**, a vocational degree and the bachelors' degree of nursing. The working group committee described **various challenges** based on both educational levels. The committee state that additional factors are important to consider when differentiating practices within the nursing profession. For example: **additional training**, specialised **educational programs** and **experience** in nurses should also be considered. Furthermore, the report stated that terminology is often ambiguous. |
| Pool et al | 2020 | Outlining the New CZO Training System (Contouren van het nieuwe CZO opleidingsstelsel) | Working group report | To describe the outlines for a prospective transition in the Dutch nursing educational system | Educational framework | This report described the insights of an independent organisation that accredits and supervises Dutch care studies. The aim of the report was to introduce a prospective outline to inform **educational** and care organisations. The outline suggested an **educational design** that is more flexible, modern, **function**-minded and **context**-specific. |

### 
